# Relationship between CAD Risk Genotype in the Chromosome 9p21 Locus and Gene Expression. Identification of Eight New *ANRIL* Splice Variants

**DOI:** 10.1371/journal.pone.0007677

**Published:** 2009-11-02

**Authors:** Lasse Folkersen, Theodosios Kyriakou, Anuj Goel, John Peden, Anders Mälarstig, Gabrielle Paulsson-Berne, Anders Hamsten, Hugh Watkins on behalf of the PROCARDIS consortia, Anders Franco-Cereceda, Anders Gabrielsen, Per Eriksson

**Affiliations:** 1 Department of Medicine, Karolinska Institute, Stockholm, Sweden; 2 Welcome Trust Centre for Human Genetics, University of Oxford, Oxford, United Kingdom; 3 Department of Molecular Medicine and Surgery, Karolinska Institute, Stockholm, Sweden; Leiden University Medical Center, Netherlands

## Abstract

**Background:**

Several genome-wide association studies have recently linked a group of single nucleotide polymorphisms in the 9p21 region with cardiovascular disease. The molecular mechanisms of this link are not fully understood. We investigated five different expression microarray datasets in order to determine if the genotype had effect on expression of any gene transcript in aorta, mammary artery, carotid plaque and lymphoblastoid cells.

**Methodology/Principal Findings:**

After multiple testing correction, no genes were found to have relation to the rs2891168 risk genotype, either on a genome-wide scale or on a regional (8 MB) scale. The neighbouring *ANRIL* gene was found to have eight novel transcript variants not previously known from literature and these varied by tissue type. We therefore performed a detailed probe-level analysis and found small stretches of significant relation to genotype but no consistent associations. In all investigated tissues we found an inverse correlation between *ANRIL* and the *MTAP* gene and a positive correlation between *ANRIL* and *CDKN2A* and *CDKN2B*.

**Conclusions/Significance:**

Investigation of relation of the risk genotype to gene expression is complicated by the transcript complexity of the locus. With our investigation of a range of relevant tissue we wish to underscore the need for careful attention to the complexity of the alternative splicing issues in the region and its implications to the design of future gene expression studies.

## Introduction

Single-nucleotide polymorphisms (SNPs) in the chromosome 9p21 region have recently been associated with cardiovascular diseases [1]–[4]. In Caucasian populations, the SNPs in question are found in a yin-yang pattern spanning 53 kb [5]. The SNPs in this haplotype block are associated with both myocardial infarction, abdominal aortic aneurysm and intracranial aneurysm[6]. A mechanism behind the link between disease and genotype is actively being sought. Recently it was reported that the expression of the nearby genes *CDKN2A* and *CDKN2B* as well as the non-coding *ANRIL* was linked with the risk genotype [7], [8]. None of the SNPs in the haplotype block are in transcribed regions, and so a change of expression level through altering of a promoter or enhancer region is indeed a plausible hypothesis.

Herein we present a detailed investigation of the relation between gene expression and the disease-associated allele at rs2891168 and other associated genotypes in a selection of relevant tissues. This SNP is a representative marker for the haplotype block.

## Results

### Analysis of Splice Variants of the ANRIL Gene

**Figure 1:**
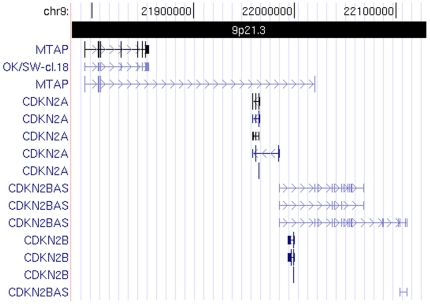
Overview of the genes of interest as annotated by the UCSC genome browser. The UCSC Genes track shows gene predictions based on data from RefSeq, Genbank, CCDS and UniProt, and is shown here to represent the current consensus on annotation. The first three references to CDKN2BAS corresponds to what is elsewhere refered to as DQ485454, EU741058 and NR_003529. They all represent different forms of the *ANRIL* gene, as taken from the literature.

RT-PCR amplification and sequencing was performed on RNA from three different cell lines and cDNA libraries in an effort to explore the different splice variants of the *ANRIL* gene. A list of the transcripts detected can be found in table 1. Full length transcripts of either 3834 or 2659 bases described previously [9] was not detected in any of the cDNA libraries investigated. However, the findings show that previously described transcripts [9] and bioinformatically annotated transcript tracks [10] do not fully cover the spectrum of alternative splicing found in *ANRIL*. Figure 1 shows the UCSC gene track as given in the UCSC genome browser as currently annotated. In this article we will refer to the gene names given in this annotation, but avoid any assumptions about the completeness of the annotation.

**Table 1:** Identified transcript variants.

Cell line	Variant identified (Exons in ANRIL)	GenBank Accesion
HUVECs	1-5-6-20	GQ495918
	1-5-6-16-18-19-20	GQ495920
	1-5-6-15-16-17-18-19-20	GQ495925
	1-5-6-7-13	EU741058
	1-5-6-7-10-13	GQ495924
Lung	1-5-6-19-20	GQ495919
	1-5-6-7-19-20	GQ495921
	1-5-6-7-16-17-18-19-20	GQ495923
Brain	1-5-6-7-16-18-19-20	GQ495922

As with the previously annotated isoforms of the *ANRIL* gene, no open reading frames longer than 100 codons were found in the new transcripts. The longest open reading frame was of codon-length 86 and was identified in the 1-5-6-7-19-20 transcript. This length is 16.3% of the transcript length and no homology for the protein sequence was identified.

### Relation between mRNA Expression Level and Risk Genotype

In the following we define the relation between a SNP and gene expression levels as the significance of an additive linear model of the expression with the genotype as effect, encoded as 0 and 2 for the homozygotes and 1 for the heterozygote.

Five datasets consisting of expression microarray data and genotypes of one or more of risk SNPs in 9p21 were analysed: two different gene expression measurements on lymphoblastoid cell lines [11], [12] from the HapMap project [13], carotid plaque tissue from the BiKE database, and medial aorta and medial mammary artery tissue from the ASAP database. The data sets investigated are further detailed in table 2.

**Table 2:** Overview of investigated data sets.

	Name	Tissue	Sample number and origin	Expression array type	Rs2891168 AA/AG/GG
**S1**	GSE9372 from hapmap cell lines	Lympoblastoid cell lines	57 CEU samples in triplicates	Affymetrix Human Exon 1.0 ST	39/93/36
**S2**	GSE7851 from hapmap cell lines	Lympoblastoid cell lines	87 CEU and 89 YRI samples	Affymetrix Human Exon 1.0 ST	20/48/19
**S3**	BiKE database	Carotid plaque tissue from endarcteromies	117 Swedish samples	Affymetrix HG-U133 plus 2.0	31/44/15
**S4**	ASAP MMed database	Medial mammary artery tissue	88 Swedish samples	Affymetrix Human Exon 1.0 ST	29/44/15
**S5**	ASAP AMed database	Medial aorta tissue	89 Swedish samples	Affymetrix Human Exon 1.0 ST	32/41/15

**Figure 2:**
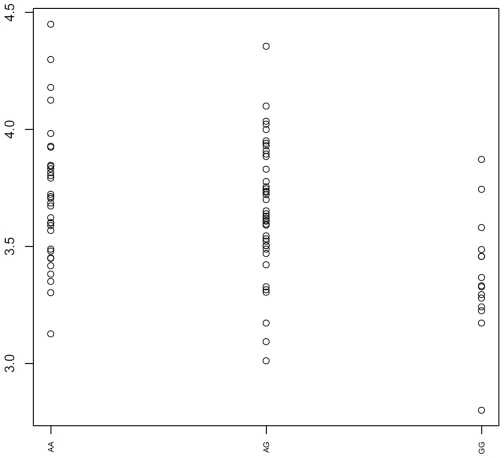
Expression plot of meta probe set 3201670 varying with rs2891168 in the ASAP AMed. Illustration of a relation between genotype and expression. The plot shows the meta probe set with Affymetrix transcript cluster ID 3201670. This probe set probes a predicted gene located 0.9 MB centromeric to rs2891168. The P-value of this relation is 1.05×10^−3^. Wen taking into account that 614 meta probe sets in the region were tested, however, this is not significant at a false discovery rate of 0.05.

Following the hypothesis that the genotype effect of rs2891168 could be observed in *trans*, ie. in genes not immediately next to rs2891168, we investigated the genome-wide set of probe sets, correcting for multiple testing at a false discovery rate of 0.05. No genes were found to significantly correlate to the genotype in any of the data sets investigated. Likewise, in a region of 8 MB centred on rs2891168 we investigated all probe sets and meta probe sets for relation to genotype. The most significant relation was found in the ASAP aorta data set. The meta probe set with Affymetrix transcript cluster ID 3201670 probing a predicted gene located 0.9 MB centromeric to rs2891168 showed a *P* = 1.05×10^−3^ relation to genotype as shown in Figure 2. However, none of the genes in the region were found to show significant correlation when correcting for multiple testing using a false discovery rate of 0.05.

On the hypothesis that rs2891168 would show a *cis*-effect, we focused on the genes within 250 KB. As indicated the splice variants in this region are not fully known for all tissue types. The regular approach of summarising microarray data into probe set and meta probe set is therefore difficult to use. Any delineation of transcripts assumes prior knowledge of transcript structure. Instead a per- probe approach was used, in which every single 25-nucleotide probe is separately analysed for relation to the genotype in question. If consecutive stretches of probes show significant relation in the same direction using the above mentioned linear model, the exons in which the probes match are likely to be part of a transcript that is regulated in relation to the genotype.

**Figure 3:**
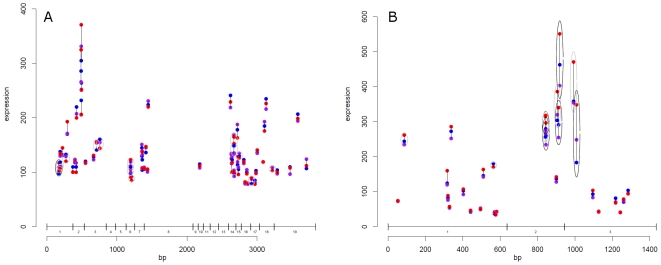
Detailed analysis of *ANRIL* in aorta tissue and *CDKN2A* in lymphoblastoid cell lines. The x-axis denotes the mRNA position in bp of the gene investigated. Exon structure of the gene is shown immediately above the axis. The y-axis shows the hybridization intensity of probes on an arbitrary scale. Each triplet of dots indicates a probe, stratified by rs2891168 genotype. Grey circles denote relation to genotype under a linear additive model at *P*<0.2. Black circles show relation at *P*<0.05. A) *ANRIL* gene with UCSC accession uc003zpm.1 in the ASAP mammary artery data set. For each probe, the median of 29 AA samples are shown as blue dots, the median of 44 AG samples are shown as purple, and 15 GG samples are shown as red. B) *CDKN2A* gene with UCSC accession uc003zpj.1 with expression data from the GSE9372 lymphoblastoid HapMap cell line data set. For each probe the median expression of 36 biological samples with the AA genotype are shown as blue, 93 AG samples as purple, and 36 GG samples in red. Samples are from triplicate growths from the same individual, and therefore represent 12, 31, and 12 different individuals. Many probes around the exon 2 and 3 boundary are found to have p-values below 0.03. The same trend is seen when summarising triplicate values as mean, and investigating per individual. These figures are available as zoomable pdf-files in supplementary material S4 and S1. Biobank materials were extracted after informed consent from all participants were obtained, according to the declaration of Helsinki and approved by the ethical committee of the Karolinska Institute, journal numbers 02-147 and 2006/784-31/1.

Figure 3A shows all unique probes matching to the *ANRIL* gene, stratified by genotype of rs2891168. Black and grey circles around each of the probe-denoting triplets indicate varying degrees of significant relations of expression to genotype. The lack of continuous stretches of significance suggests that no single part of *ANRIL* has expression related to rs2891168 in the ASAP mammary artery data set. Similar investigations were performed for *MTAP*, *CDKN2A*, *CDKN2B* and *ANRIL*. Isoforms containing all UCSC known exons were used as template sequences for the analysis, and differences should therefore be observable both in previously identified isoforms (p15^INK4b^, p16^INK4a^, ARF etc.) as well as in hitherto unidentified splice variants.

Using this fine grained analysis model all data sets specified in table 2 were investigated. The full result is included as supplementary files S1, S2, S3, S4, and S5. The longest continuous stretch of significant probes was in the *CDKN2A* gene, in a region in the exon 2 to 3 junction of the *CDKN2A* variant known as uc003zpj.1 in the UCSC genome browser. This was found in the lymphoblastoid cell line of the GSE9372 data set and is shown in Figure 3B. Eight of nine probes in the region show a trend of relation to rs2891168, five probes at *P*<0.05. The GSE7851 data set, which measures another growth of the HapMap lymphoblastoid cell lines, did not show this relation. In the BiKE database the investigation was also performed using only samples from the two main complementary haplotypes previously described [5]. Results were largely similar to findings when only the rs2891168 was investigated, so haplotype analysis was not further pursued.

#### Co-Expression Studies

All pair wise combinations of genes in an 8 MB region centred on rs2891168 were investigated for correlation of expression levels. This was done to determine if longer range interactions between genes was in effect, in particular for pairings with one of the genes localised close to rs2891168. The strongest long-distance correlation found was between *MTAP* and a gene *DENND4C* found 2.7 MB telomeric to rs2891168. This pair showed a Pearson correlation coefficient of 0.847 in the ASAP AMed data and 0.810 in the ASAP MMed data set. These correlations had *P*<10^−16^, which was significant after correcting for multiple testing on 1596 pairings. There was no correlation between the previously discussed predicted gene with Affymetrix ID 3201670 and any of the genes in the immediate vicinity of rs2891168. Supplementary file S6 shows a complete overview of coexpression in the region.

**Figure 4:**
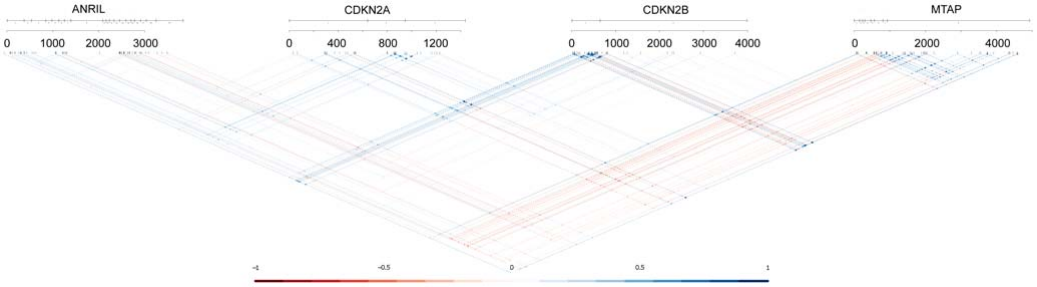
Co-expression of *ANRIL*, *CDKN2A*, *CDKN2B*, and *MTAP* in aorta tissue. Correlation between all pairwise combinations of microarray probes in *ANRIL*, *CDKN2A*, *CDKN2B*, and *MTAP*. Dots at the intersection between two probes indicate correlation, in the same style as a Haploview plot. The colour and the size of each dot are dependent on the Pearson correlation coefficient, with the colour-scale on the bottom of the figure giving the values. For each gene, the X-axis gives the location of a given probe in the mRNA in nucleotides. See reference [14] for further explanation. Data is taken from the ASAP aorta database, but the trend that *MTAP* shows inverse correlation with the three other genes is general for all data sets. Likewise, some degree of positive correlation between *ANRIL*, *CDKN2A* and *CDKN2B* is observed in all data sets.

In the region of *MTAP*, *CDKN2A*, *CDKN2B* and *ANRIL* co-expression was investigated using probe-level visualization tools from GeneRegionScan [14]. This allows analysis of all possible probe pair combinations within a given local region, thereby avoiding the problem of strictly defining transcript structure. A striking feature in all of the data sets analysed is that *MTAP* expression, especially the seven exons most 5′, shows inverse correlation with *ANRIL*, *CDKN2A*, and in particular *CDKN2B* expression. The positive correlation between *CDKN2A*, *CDKN2B* and *ANRIL* that has previously been reported [9], was also found in the data sets under investigation here. To what degree depends on the data set, with the clearest effect seen in the ASAP aorta data set as shown in Figure 4.

While not generally used as quality control measure in microarray analysis, the correlation of the intensity of probes within the same exon can be used to estimate noise levels. Without any noise in the measurement it would be expected that these probes show perfect correlation. Following this, it should be noted that some of the probes with low expression, show close to no correlation with neighbouring probes, particularly in the *ANRIL* region. The low expression of *ANRIL* places it close to the detection limit. Some degree of detection power does remain because we accurately detect changes from the rs10965215 SNP, a SNP that is found in the middle of the first probe of exon 2. This SNP is linked with rs2891168 (D′ = 0.603, R^2^ = 0.34) in the CEU population [15], and will affect the hybridization efficiency of the probe because of the sequence change. This is in fact observed in the CEU population data sets, and we conclude that the microarray measurements have at least some degree of detection power at the expression levels of *ANRIL*.

## Discussion

Recent studies have searched for links between the genotypes from the described yin-yang haplotype block and expression levels of nearby genes. At the time of publication, these were the studies by Jarinova et al. [8] and Liu et al. [7].

One finding of the Jarinova et al. study is that the risk allele is linked to expression levels of ANRIL in whole blood cells from healthy human subjects. The direction of this regulation is dependent on which transcript variant is measured [8]. The Liu et al. study suggests that the risk allele is linked to decreased expression of *CDKN2A*, *CDKN2B* and *ANRIL* in peripheral blood T-cells. This was identified in T-cells from 170 healthy donors. It is not known in which exons these expression measurements are performed [7].

The rs10757278 SNP investigated in the Liu et al. study and the rs1333045 SNP investigated in the Jarinova et al. study are both found in the same haplotype block as the rs2891168 SNPs, with which they are in strong LD (r^2^ = 0.894 and r^2^ = 0.746) in the HapMap CEU population [15]. However, the observed relations were seen neither in lymphoblastoid cell lines from 87 healthy CEU individuals nor in carotid plaque tissue from 117 patients undergoing endarcteromy and in aorta and mammary artery tissue from 89 patients undergoing treatment for valve disease. On the contrary, one of the HapMap data sets, the GSE9372 set, revealed some degree of relation between increased expression of *CDKN2A* and the risk allele of the rs2891168.

One possible reason for this contradiction is the size of the data sets. It is possible that this investigation does not have the power to identify the associations found. However, in four different data sets of approximately half the size of the sets presented by Liu et al.[7], no trends supporting a general expression decrease were observed. The sample size of Jarinova et al. [8] is comparable to this study.

Another reason could be the different tissue types investigated. Liu et al. investigates T-cells and Jarinova et al. investigates whole blood cell. It is possible that gene expression changes are only observed in immune cells, and that immune cells are central to the mechanisms of cardiovascular disease affected by rs2891168 and its linkage block. Indeed, the inflammatory response system is known to play a central role in central aspects of cardiovascular pathogenesis. However, the block has been linked to other disease patterns that are thought to be independent of immune response, in particular intracranial aneurysm [6]. In light of this we would expect the effect of the genotype to be found in tissue types that are implicated in all disease processes to which it has been linked.

One major issue that is likely to be central is that alternative splicing events are masking reported results. We found splicing events in *ANRIL* to be more extensive than previously reported. We identified 8 new *ANRIL* splice variants but did not detect the short and long form splice variants. While this is not certain to cover the full spectrum of all possible splice variants in all tissues, it highlights the fact that more extensive alternative splicing does take place and will affect conclusions made without knowledge of isoform architecture. The original description of ANRIL reported the short and long splice variants (2659 and 3834 bp respectively) in normal human testis mRNA. In the same report, *ANRIL* expression in a range of other tissues was detected using PCR with primers specific to exons 14–16. It is possible therefore that the expression of the previously annotated short and long form is tissue specific [9]. Thus, any expression analysis of *ANRIL* that is dependent on the presence of specific transcript variants should undertake a systematic survey of the alternative splice variants present in each sample type.

We present these results to underscore the fact that the region surrounding rs2891168 and its linkage block has a complex pattern of variable splicing and expression change, and that any conclusions should be made cautiously. It is important to emphasize that future studies investigating expression of *ANRIL* should take into account the presence of multiple transcript variants that may also vary between different tissue types.

## Materials and Methods

### Ethics Statement

Biobank materials were extracted after informed consent from all participants were obtained according to the declaration of Helsinki and approved by the ethical committee of the Karolinska Institute, journal numbers 02-147 and 2006/784-31/1.

### Sample Collection

The ASAP study included patients undergoing heart-valve surgery at Karolinska University Hospital, Stockholm, Sweden. Biopsies were obtained at surgery from dilated and non-dilated ascending aorta and from mammary artery. The medial layer was isolated by adventicectomy, incubated with RNAlater (Ambion) and homogenized with a FastPrep (Qbiogene, Irvine, CA) using Lysing Matrix D tubes (Invitro cat.no. 6913-100). The BiKE study included atherosclerotic tissue collected from patients undergoing carotid endarterectomy at Karolinska University Hospital, Stockholm, Sweden. For both biobanks, total RNA was isolated using Trizol (BRL-Life Technologies) and RNeasy Mini kit (Qiagen) as a cleanup including treatment with RNase-free DNase set (Qiagen) according the manufacturer's instructions. The quality of RNA was analyzed with an Agilent 2100 bioanalyzer (Agilent Technologies Inc., Paolo Alto, CA, USA) and quantity was measured by a NanoDrop (Thermo Scientific).

### PCR Analysis of Splice Variants

A forward primer annealing on *ANRIL* exon 1 found in all described alternative transcripts (Exon1F ctcgtcgaaagtcttccattct) and reverse primers annealing on exon 13 of the shorter transcript (Exon13R caagatagagaagcaggtatc) and exon 19 of the longer transcript (Exon19R atacagaaagatgaaaagtgatttgcc) were employed to amplify full length transcripts as described [9]. The Exon13R primer was used only in the analysis of HUVEC cells. PCR included 35 cycles of a denaturation step for 15 sec followed by annealing at 55°C for 15 seconds and a polymerase extension step of 72°C for 3 minutes. PCR products were run on 1% agarose gel and products extracted using the QIAquick gel extraction kit (Qiagen). Purified products were sequenced by capillary sequencing.

### Expression Microarray Processing

The BiKE and ASAP RNA samples were hybridized and scanned on the Karolinska Institute microarray core facility. For samples from the BiKE biobank Affymetrix HG-U133 plus 2.0 arrays and protocols were used, whereas Affymetrix GeneChip® Human Exon 1.0 ST arrays and protocols were used for samples from the ASAP biobank. Cel files from the GSE9372 and GSE7851 dataset were downloaded from the Gene Expression Omnibus [16].

For probe set and meta probe set level investigations, ie. the genome-wide and regional investigations, cel files were pre-processed using Robust Multichip Average (RMA) [17] normalization as implemented in the Affymetrix Power Tools 1.8.6 package apt-probeset-summarize. Using HG-U133 plus 2.0 arrays all probe sets were included, but when using Exon 1.0 ST arrays the investigation was limited to the core subset of well-validated meta probe sets, in the genome-wide scan. In the regional scan, the full set of meta probe sets was used.

Local investigations of the *MTAP*, *CDKN2A*, *CDKN2B* and *ANRIL* genes were done using the GeneRegionScan package [14] from the Bioconductor repository [18] to extract probe level data and de novo annotate each probe to its location along the length of the gene.

### Statistics

P-values are presented as unmodified p-values, however multiple testing corrections were performed as stated in the text using the qvalue package [19], using a false discovery rate cut-off of 0.05.

The triplicates of GSE9372 were treated as individual data points, because they represent different biological growths of each cell line. Parallel investigations were made with the data summarised per individual using the mean values, but results where similar (data not shown).

### Genotyping

Genotypes for the GSE9372 and GSE7851 data sets were obtained from the HapMap project webpage [15]. For ASAP and BIKE, genomic DNA was isolated from peripheral blood leukocytes using the QIAamp® DNA Mini Kit (Qiagen, Germany). Genotypes were measured using a TaqMan Allelic discrimination method (Perkin Elmer Biosystems, Foster city, CA, USA). In the ASAP data set, only the rs2891168 was measured. In the BiKE data set the additional SNPs rs2383207, rs2383206, rs1333049, rs1333045, rs10757278, rs10757274, and rs10116277 were genotyped in order to investigate the two main complementary CAD-risk haplotypes.

## Supporting Information

File S1 Detailed local analysis in GSE9372 data set Top half of plot shows expression level of individual microarray probes as a function of location on gene. The four genes ANRIL, CDKN2A, CDKN2B and MTAP are analysed. Triplets of dots indicate median values of samples that are AA, AG and GG respectively. Bottom half of plot shows Pearson correlation between all pairwise combinations of probes analysed.
(0.33 MB PDF)

File S2 Detailed local analysis in GSE7851 data set Top half of plot shows expression level of individual microarray probes as a function of location on gene. The four genes ANRIL, CDKN2A, CDKN2B and MTAP are analysed. Triplets of dots indicate median values of samples that are AA, AG and GG respectively. Bottom half of plot shows Pearson correlation between all pairwise combinations of probes analysed.
(0.38 MB PDF)

File S3 Detailed local analysis in BiKE data set Top half of plot shows expression level of individual microarray probes as a function of location on gene. The four genes ANRIL, CDKN2A, CDKN2B and MTAP are analysed. Triplets of dots indicate median values of samples that are AA, AG and GG respectively. Bottom half of plot shows Pearson correlation between all pairwise combinations of probes analysed.
(0.29 MB PDF)

File S4 Detailed local analysis in ASAP MMed data set Top half of plot shows expression level of individual microarray probes as a function of location on gene. The four genes ANRIL, CDKN2A, CDKN2B and MTAP are analysed. Triplets of dots indicate median values of samples that are AA, AG and GG respectively. Bottom half of plot shows Pearson correlation between all pairwise combinations of probes analysed.
(0.42 MB PDF)

File S5 Detailed local analysis in ASAP AMed data set Top half of plot shows expression level of individual microarray probes as a function of location on gene. The four genes ANRIL, CDKN2A, CDKN2B and MTAP are analysed. Triplets of dots indicate median values of samples that are AA, AG and GG respectively. Bottom half of plot shows Pearson correlation between all pairwise combinations of probes analysed.
(0.45 MB PDF)

File S6 Analysis of coexpression in 8 MB region around rs2891168 Pearson correlation between all pairwise combinations of genes in a 8 MB region. All genes found in the full subset of affymetrix meta probe sets were analysed, but only genes with a genesymbol are shown here for clarity. This produces an all most similar plot, because the more putative genes show little correlation.
(0.20 MB PDF)
