# Supplementary figures and images for: Relationship between CAD Risk Genotype in the Chromosome 9p21 Locus and Gene Expression. Identification of Eight New ANRIL Splice Variants
Source: PLoS One. 2009 Nov 2;4(11):e7677. doi: 10.1371/journal.pone.0007677 (PMC2765615; doi:10.1371/journal.pone.0007677)

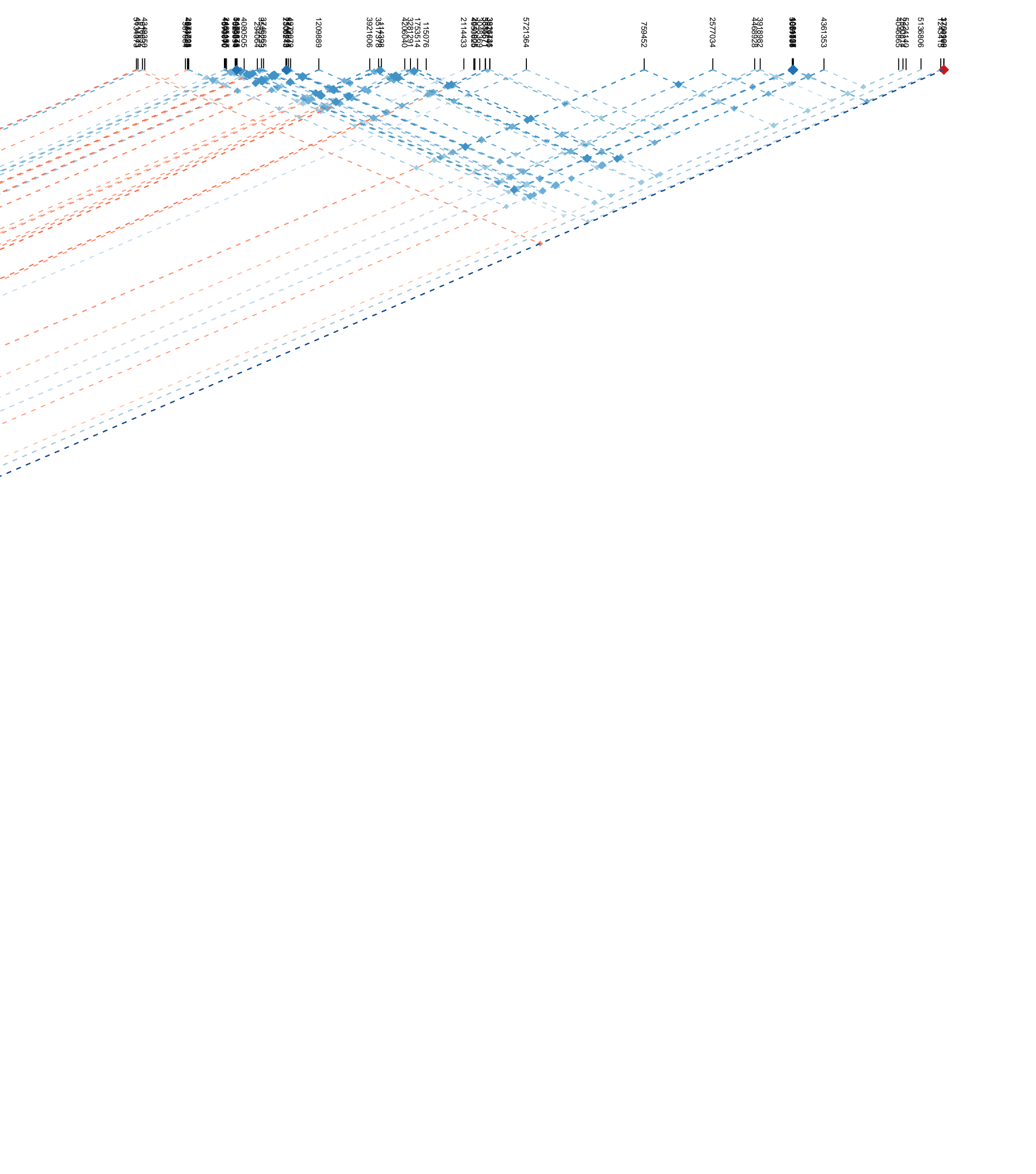

Supplement: File S1 — Detailed local analysis in GSE9372 data set Top half of plot shows expression level of individual microarray probes as a function of location on gene. The four genes ANRIL, CDKN2A, CDKN2B and MTAP are analysed. Triplets of dots indicate median values of samples that are AA, AG and GG respectively. Bottom half of plot shows Pearson correlation between all pairwise combinations of probes analysed. (0.33 MB PDF) [file pone.0007677.s001.pdf]

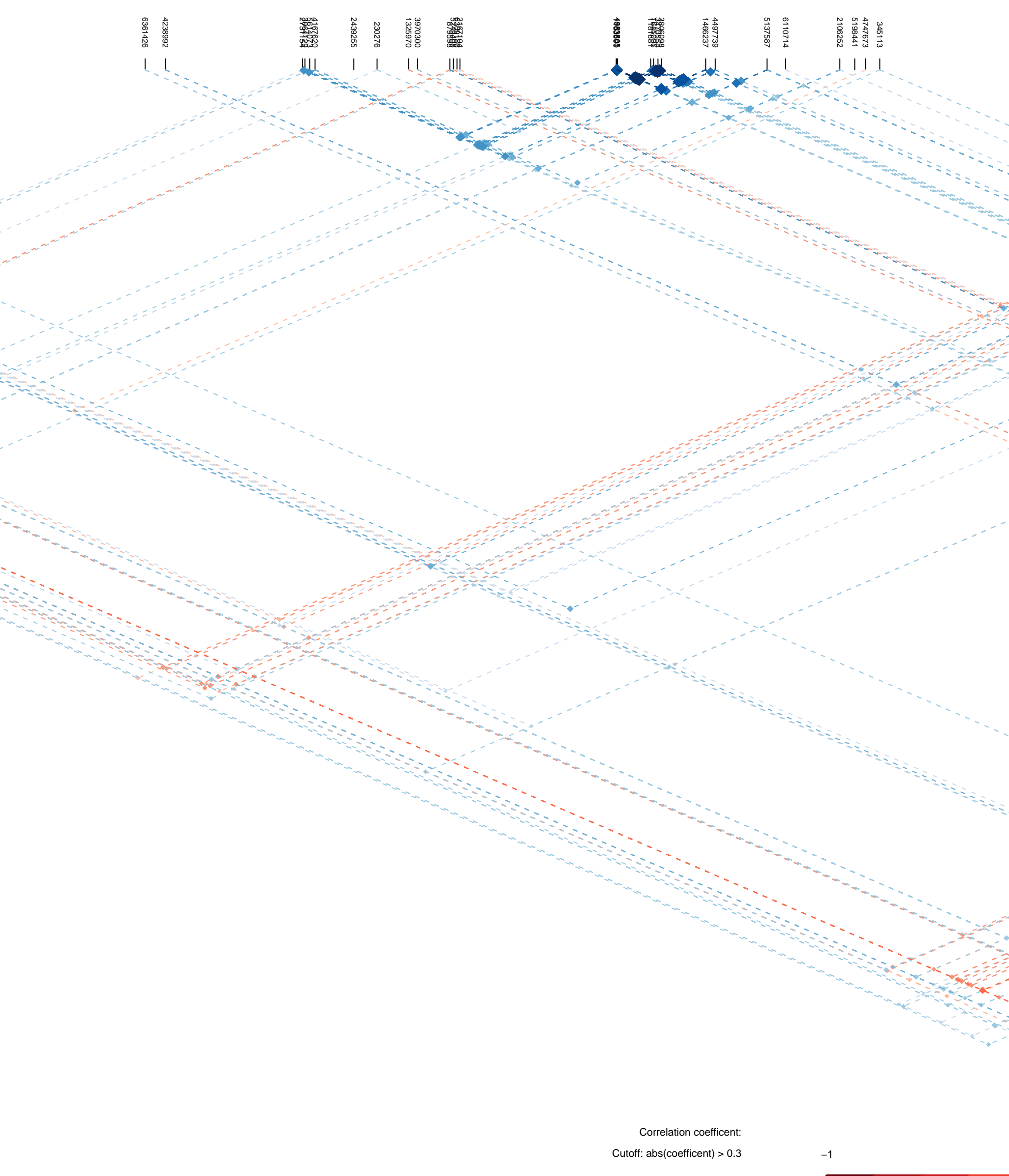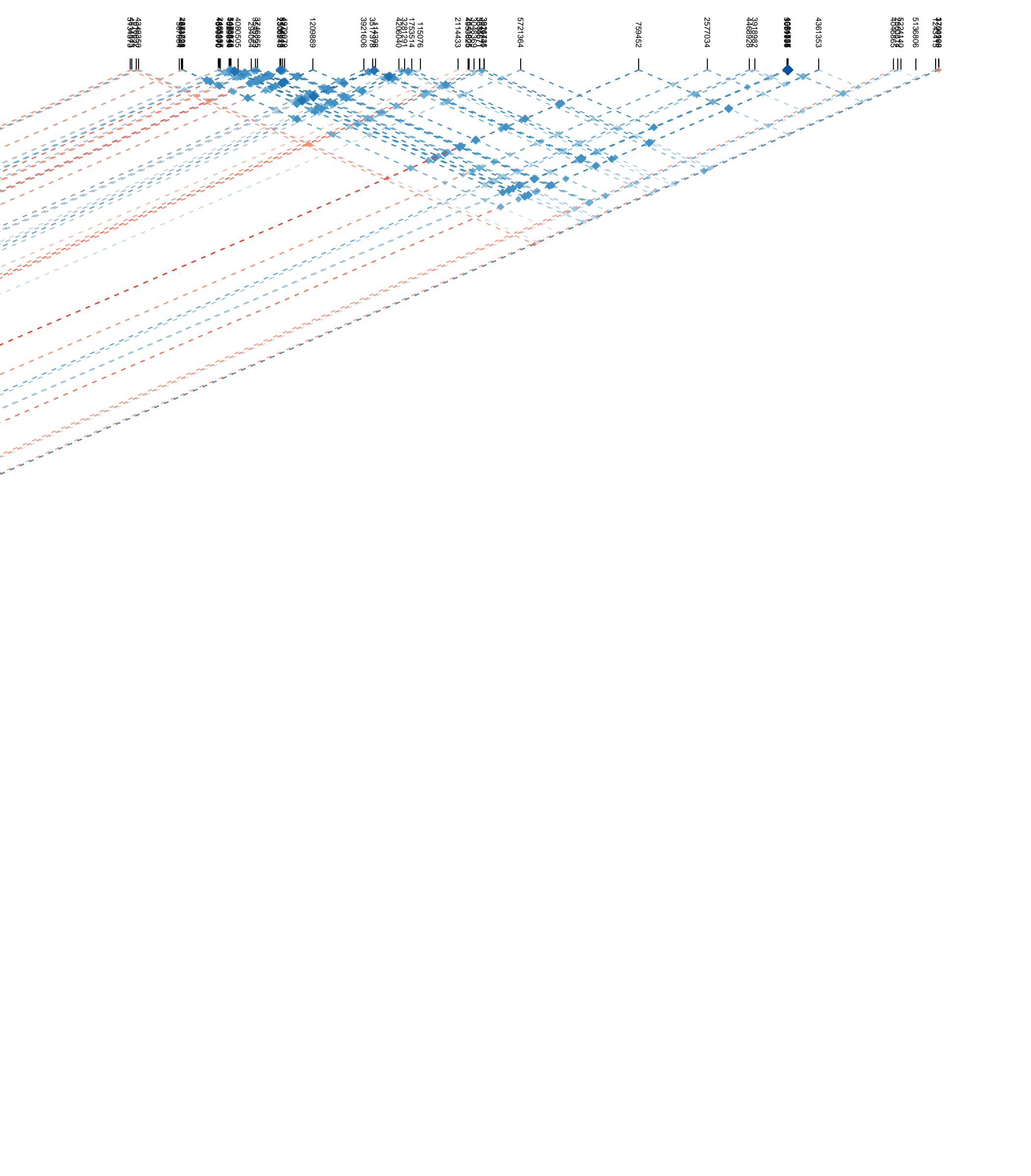

Supplement: File S2 — Detailed local analysis in GSE7851 data set Top half of plot shows expression level of individual microarray probes as a function of location on gene. The four genes ANRIL, CDKN2A, CDKN2B and MTAP are analysed. Triplets of dots indicate median values of samples that are AA, AG and GG respectively. Bottom half of plot shows Pearson correlation between all pairwise combinations of probes analysed. (0.38 MB PDF) [file pone.0007677.s002.pdf]

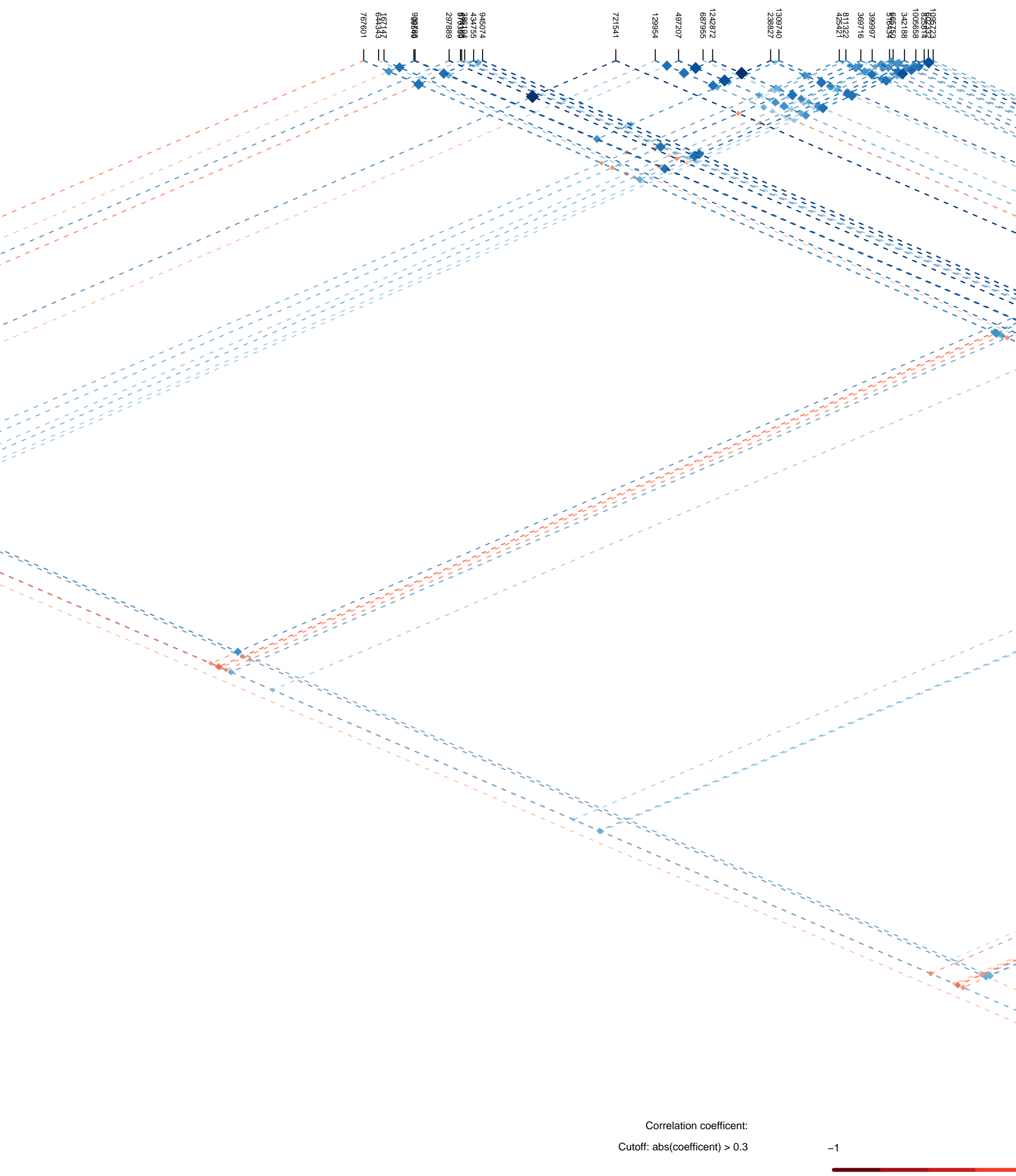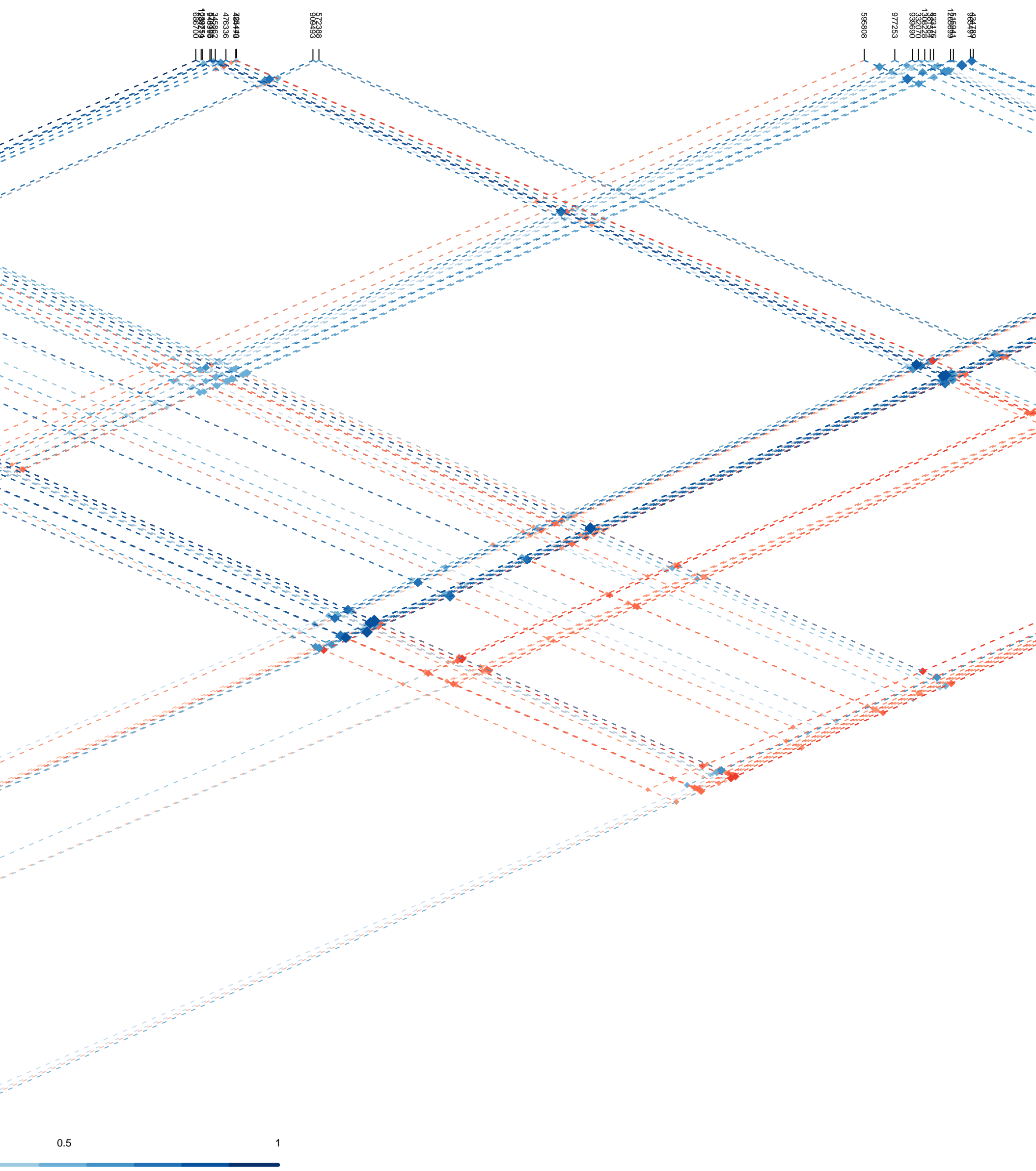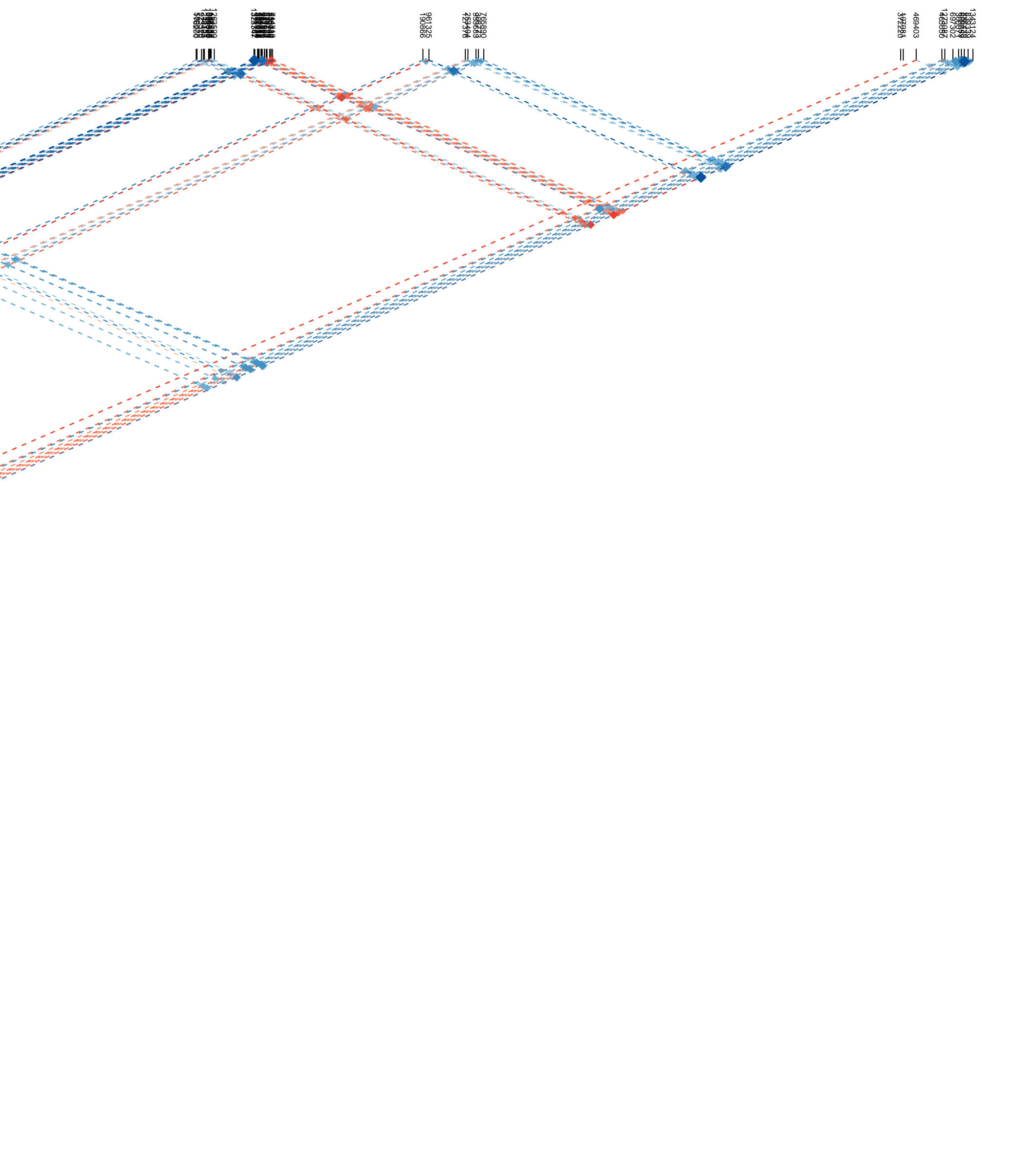

Supplement: File S3 — Detailed local analysis in BiKE data set Top half of plot shows expression level of individual microarray probes as a function of location on gene. The four genes ANRIL, CDKN2A, CDKN2B and MTAP are analysed. Triplets of dots indicate median values of samples that are AA, AG and GG respectively. Bottom half of plot shows Pearson correlation between all pairwise combinations of probes analysed. (0.29 MB PDF) [file pone.0007677.s003.pdf]

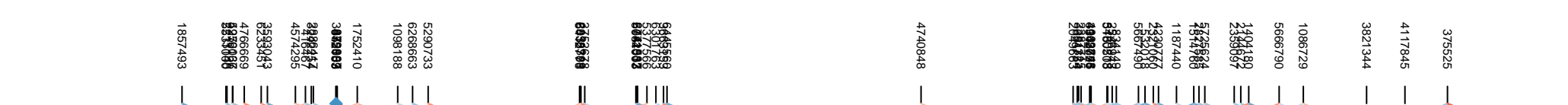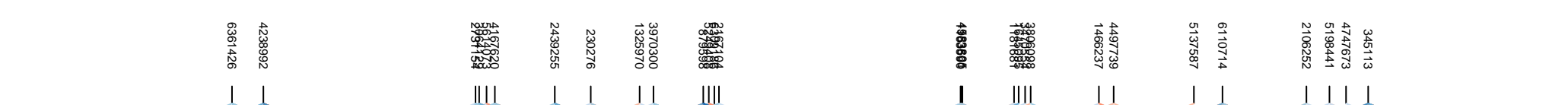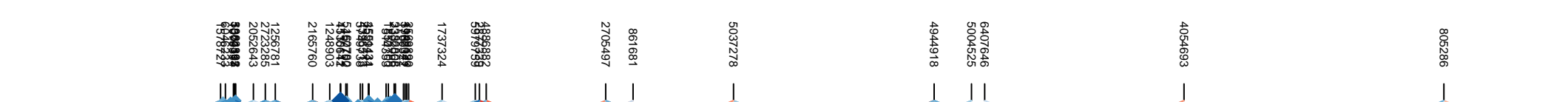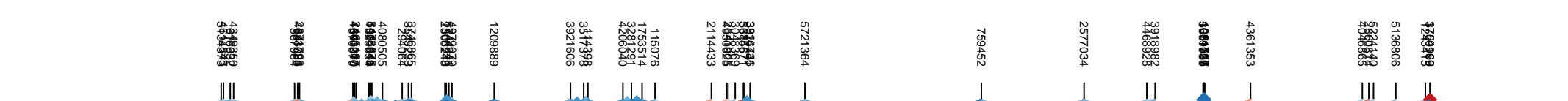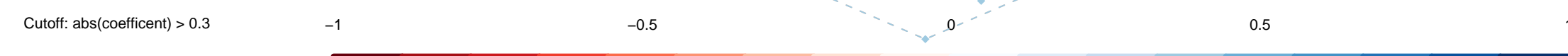

Supplement: File S4 — Detailed local analysis in ASAP MMed data set Top half of plot shows expression level of individual microarray probes as a function of location on gene. The four genes ANRIL, CDKN2A, CDKN2B and MTAP are analysed. Triplets of dots indicate median values of samples that are AA, AG and GG respectively. Bottom half of plot shows Pearson correlation between all pairwise combinations of probes analysed. (0.42 MB PDF) [file pone.0007677.s004.pdf]

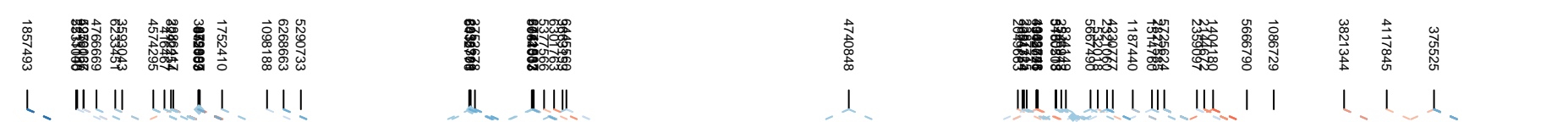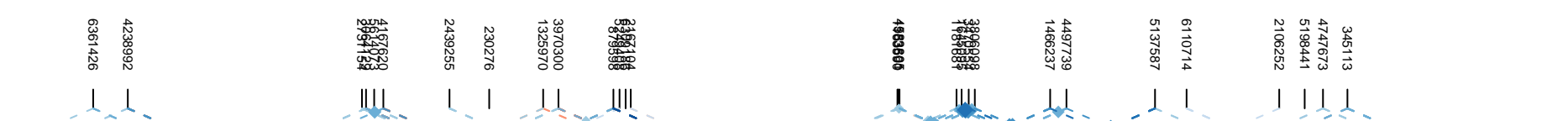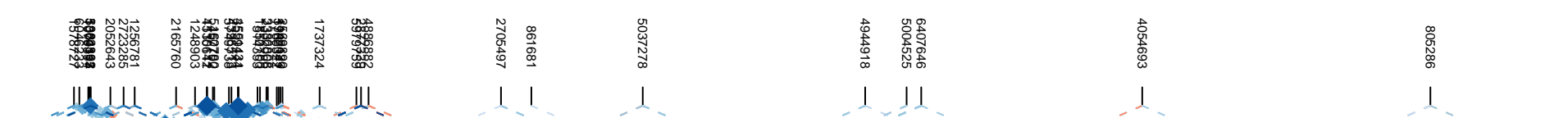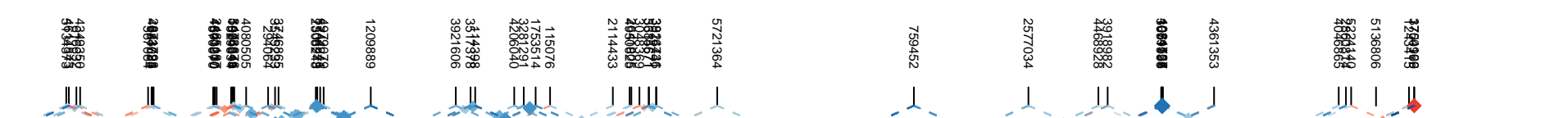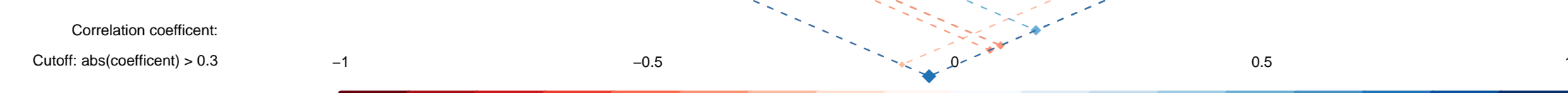

Supplement: File S5 — Detailed local analysis in ASAP AMed data set Top half of plot shows expression level of individual microarray probes as a function of location on gene. The four genes ANRIL, CDKN2A, CDKN2B and MTAP are analysed. Triplets of dots indicate median values of samples that are AA, AG and GG respectively. Bottom half of plot shows Pearson correlation between all pairwise combinations of probes analysed. (0.45 MB PDF) [file pone.0007677.s005.pdf]

1

0

month,

ation.

-1

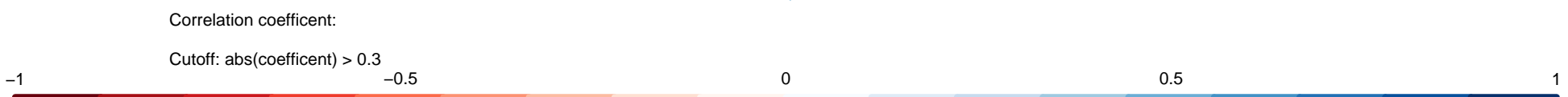

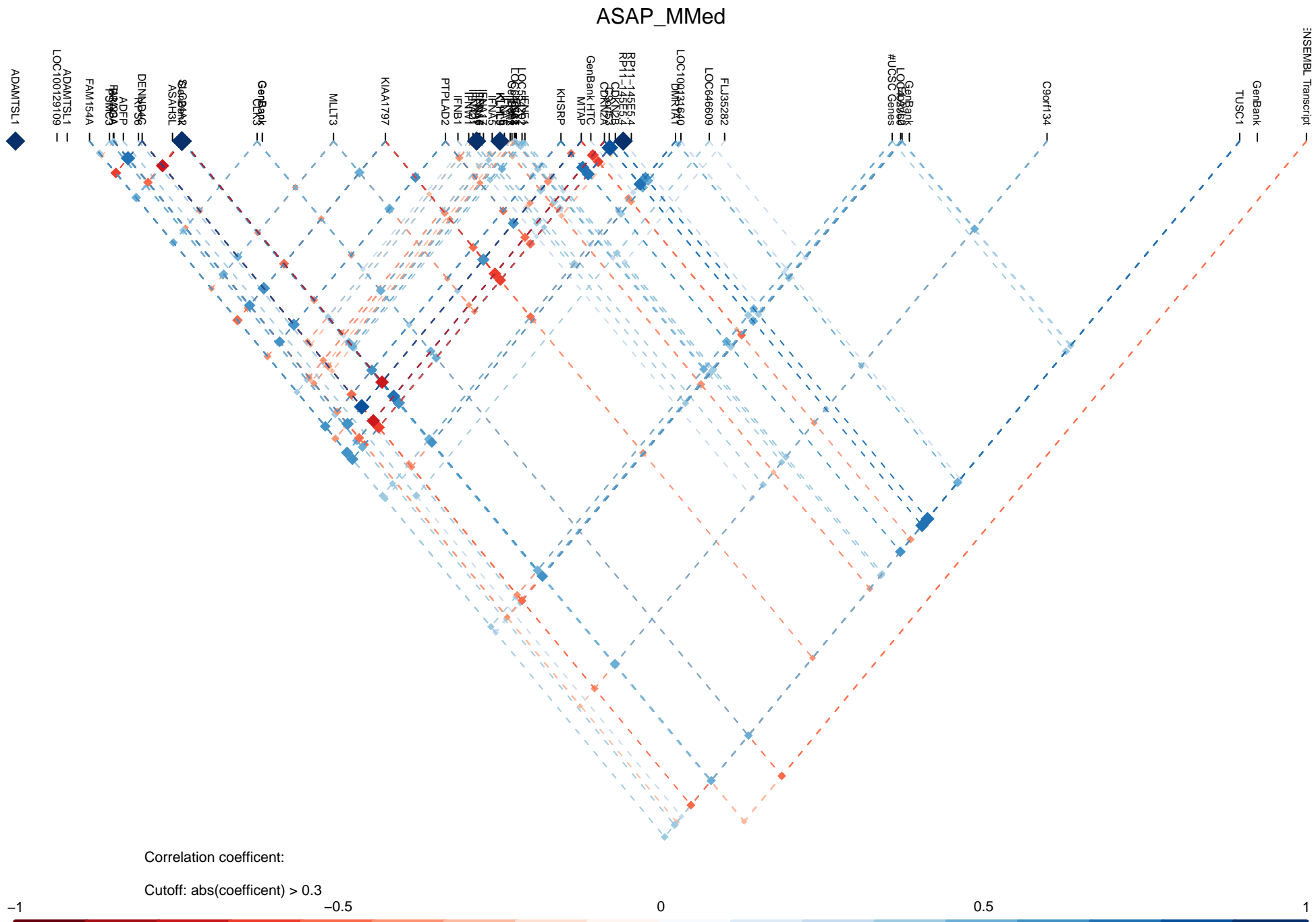

## GSE7851\_ceu\_only

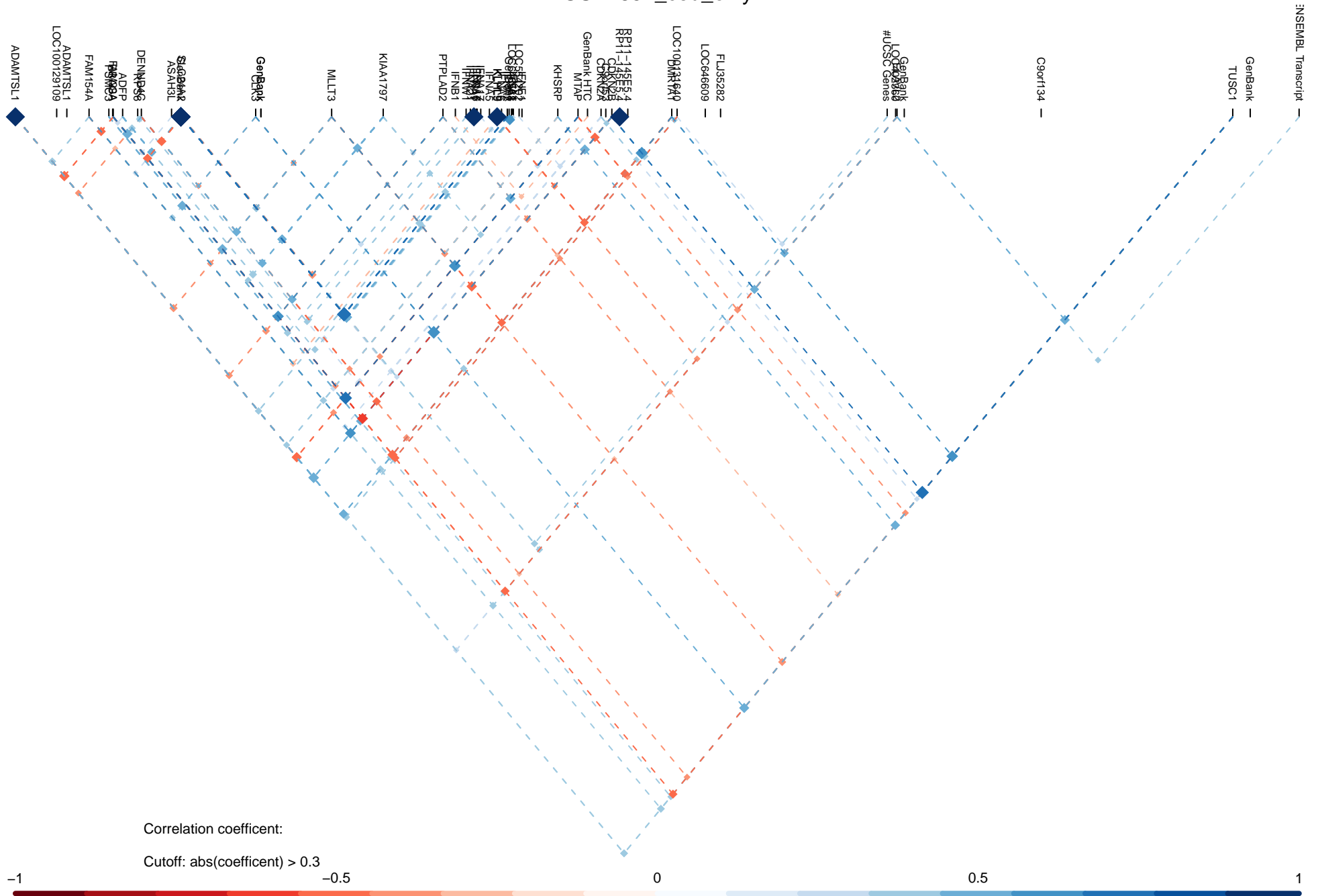

1

(

-0.5

 $\sin(\theta) = \frac{y}{r}$ 

(b)(6)

con. a

-1

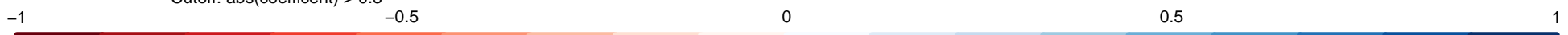

Supplement: File S6 — Analysis of coexpression in 8 MB region around rs2891168 Pearson correlation between all pairwise combinations of genes in a 8 MB region. All genes found in the full subset of affymetrix meta probe sets were analysed, but only genes with a genesymbol are shown here for clarity. This produces an all most similar plot, because the more putative genes show little correlation. (0.20 MB PDF) [file pone.0007677.s006.pdf]
